# Catarrh of the Bladder

**Published:** 1880-06

**Authors:** 


Catarrh of the Bladder.—There is a group of symptoms frequently met with in men of advancing years, to which I desire especially to call your attention. One of the first circumstances to attract notice is that the urine is more or less cloudy when passed, and that on standing it deposits some adhesive opaque matter in the bottom of the vessel. Such urine is generally neutral, or at best faintly acid; occasionally it is alkaline, always becoming so rapidly by keeping. On interrogating the patient, you learn that the act of micturition is performed rather more frequently than natural—that he is disturbed by it two or three times in the night, and every two hours or so during the day. He may also complain of dull pains about the pelvis and back; he finds the effort to pass water rather greater than it formerly was, and the general health has of late suffered a little.

Now, it is by no means uncommon to hear this group of symptoms spoken of as indicating the presence of a "catarrh of the bladder." And catarrh of the bladder is very generally regarded as a particularly obstinate, sometimes indeed as an incurable, affection. And I am free to confess that as long as these phenomena are considered referable to a specific disease, "catarrh," so long probably will the disease prove rebellious to treatment and sometimes even incurable.

Nothing is more common than to hear, in connection with these cases, the remark gravely and significantly made: "I assure you I have on several occasions found by testing, a large quantity of albumen in the patient's urine." Does the observer really desire to intimate that the patient has constitutional albuminuria—*i. e.*, some form of Bright's disease? If not, his remark is simply devoid of meaning; since, as we all know, there is vesical pus in the urine, we know equally that the albuminous constituent must appear on applying the test. And vesical pus in the urine certainly has no more relation to constitutional albuminuria than pus which comes from an external abscess or surrounds a common boil. Simple as all this may appear to you and me, it is quite astonishing how much confusion there is in men's minds in regard to this matter, and how much importance some attach to all albumen found in a urinary test-tube, although the source of the deposit may be easily demonstrated to be the bladder, and no other part of the organs which lie above it.

The important practical point in relation to treatment is first to ascertain the occasion of the local catarrh. In nine out of ten of these cases it consists in inability, often only to a slight extent, on the part of the patient to empty the bladder completely. The universally acknowledged cause, hypertrophy of the prostrate, is of course, the first in order of frequency. But after this are others not infrequent. Defective action may be due, first, to simple atony, the result of past habitual or occasional over-distension of the bladder with urine; secondly, to thickening and incompetent muscular action of the bladder after chronic inflammation, sometimes associated with old stricture; thirdly, to defective innervation seen in connection with other slight signs of impaired function in a nervous centre, the last being, of course, the most serious of all, in its nature and probable results.

In all of these local treatment, by carefully removing all the secretion by means of a soft catheter two or three times a day, perhaps aided by gently washing out the remainder, is the chief efficient remedy. Remember that this incompetency of the bladder is always to be sought for by physical examination; no other form of evidence in relation to it, as the patient's sensation, etc., is to be accepted as trustworthy. The introduction of a soft catheter immediately after the patient has passed water by its natural efforts, is the only test, and it should be applied on two or three occasions before arriving at a definite conclusion. The casual relation between the group of symptoms enumerated at the outset, and the defective function described, is far more common than it is generally supposed to be. It is on this account, therefore, that I have asked your attention specially to the subject—*Sir Henry Thompson in London Lancet.*

[We would suggest that the wash be made astringent or slightly cathartic.—Ed. A. M. and S. J.]

